# Valproic Acid Protects Primary Dopamine Neurons from MPP^+^-Induced Neurotoxicity: Involvement of GSK3*β* Phosphorylation by Akt and ERK through the Mitochondrial Intrinsic Apoptotic Pathway

**DOI:** 10.1155/2017/8124501

**Published:** 2017-03-22

**Authors:** Chi Zhang, Xianrui Yuan, Zhongliang Hu, Songlin Liu, Haoyu Li, Ming Wu, Jian Yuan, Zijin Zhao, Jun Su, Xiangyu Wang, Yiwei Liao, Qing Liu

**Affiliations:** ^1^Department of Neurosurgery, Xiangya Hospital, Central South University, Changsha, Hunan 410008, China; ^2^Department of Pathology, Xiangya Hospital, Central South University, Changsha, Hunan 410008, China

## Abstract

Valproic acid (VPA), a drug widely used to treat manic disorder and epilepsy, has recently shown neuroprotective effects in several neurological diseases, particularly in Parkinson's disease (PD). The goal of the present study was to confirm VPA's dose-dependent neuroprotective propensities in the MPP^+^ model of PD in primary dopamine (DA) neurons and to investigate the underlying molecular mechanisms using specific mitogen-activated protein kinases (MAPKs) and phosphatidylinositol 3-kinase- (PI3K-) Akt signaling inhibitors. VPA reversed MPP^+^-induced mitochondrial apoptosis and counteracted MPP^+^-induced extracellular signal-regulated kinase (ERK) and Akt repression and inhibited glycogen synthase kinase 3*β* (GSK3*β*) activation through induction of GSK3*β* phosphorylation. Moreover, inhibitors of the PI3K and MAPK pathways abolished GSK3*β* phosphorylation and diminished the VPA-induced neuroprotective effect. These findings indicated that VPA's neuroprotective effect in the MPP^+^-model of PD is associated with GSK3*β* phosphorylation via Akt and ERK activation in the mitochondrial intrinsic apoptotic pathway. Thus, VPA may be a promising therapeutic candidate for clinical treatment of PD.

## 1. Introduction

Parkinson's disease (PD) is a chronic and progressive disorder of the nervous system that affects nearly one million people and causes an economic burden of nearly $25 billion per year in the United States alone [1, 2]. PD affects the patient's movement with the cardinal motor symptoms of resting tremor, bradykinesia, freezing of gait, and rigidity [3]. The motor manifestations of PD are attributable to the degeneration and decrease in the number of dopamine-generating cells in the substantia nigra pars compacta (SNpc) [4]. Although a variety of cellular and molecular changes, such as oxidative stress, mitochondrial dysfunction, and endoplasmic reticulum (ER) stress, have been implicated in the pathophysiology of PD, the etiology of the selective loss of dopaminergic neurons in SNc has not been fully elucidated [4–6]. Emerging evidence showed that apoptosis plays a fundamental role in PD's pathology. Therefore, therapeutic strategies aimed at providing neuroprotective effects against apoptosis may be beneficial in the treatment of PD [7, 8].

Valproic acid (VPA, 2-propylpentanoic acid), a short branched chain fatty acid, has been used worldwide in the treatment of epilepsy and bipolar disorder for decades [9]. In addition, VPA has shown effects on neurotransmission adjustment and intracellular pathway modulation during cell growth, differentiation, and apoptosis [10]. VPA has neuroprotective properties in several neurological diseases, particularly in PD. VPA treatment significantly counteracted the death of nigral neurons in vivo and in vitro [11–14]. Previous studies suggested that multiple signaling pathways are associated with PD pathology, including phosphoinositide 3-kinase (PI3K), mitogen-activated protein kinases (MAPKs), and other pathways, which may be regulated by VPA [15–18]. Nonetheless, it is not fully understood how VPA interacts with these pathways and causes neuroprotection. Interestingly, PI3K and MAPK pathways can both regulate downstream glycogen synthase kinase 3*β* (GSK3*β*), which is directly linked to the mitochondrial intrinsic apoptosis pathway [19, 20]. However, whether GSK3*β*-related mitochondrial intrinsic apoptosis contributes to VPA-induced neuroprotection in PD has not been clearly determined.

Our present study explored VPA's neuroprotective propensities in the MPP^+^-induced PD model in primary cultured dopamine (DA) neurons and focused on the role of VPA in the GSK3*β*-activated mitochondrial apoptosis pathway by using PI3K and MAPK pathway-specific inhibitors.

## 2. Results

### 2.1. VPA Dose-Dependently Protected Dopamine Neurons in MPP^+^-Induced Neurotoxicity

**Figure 1:**
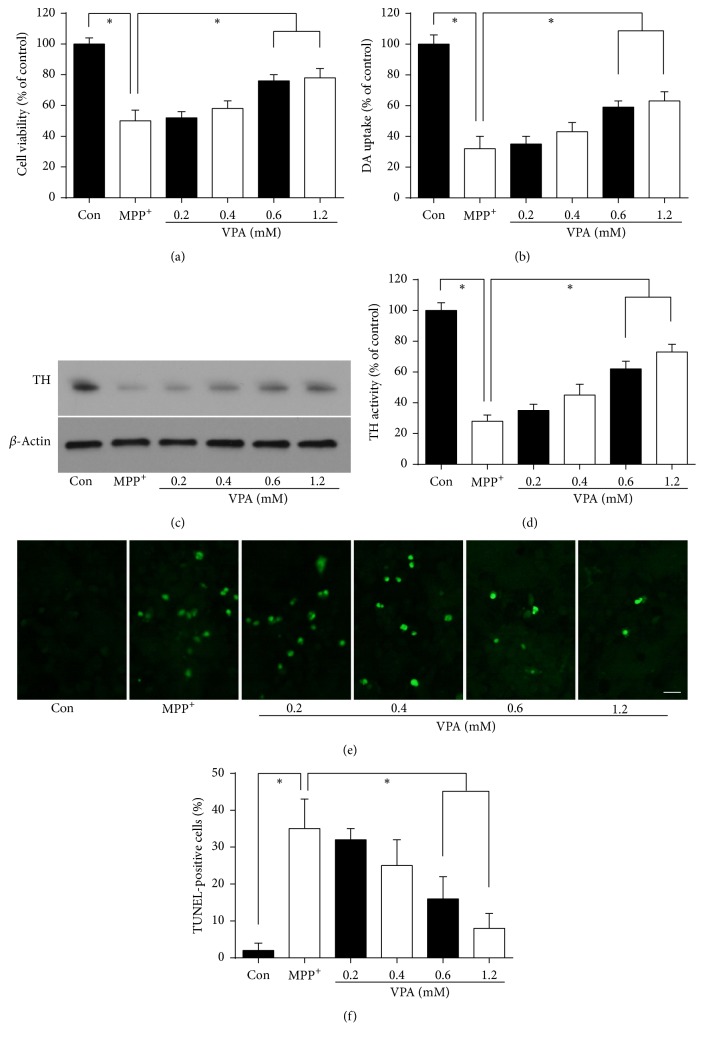
VPA dose-dependently protected DA neurons against MPP+-induced neurotoxicity. Different doses of VPA (0.2, 0.4, 0.6, and 1.2 mM) were given to cultured DA neurons after treatment with 10 *µ*M MPP+ for 48 h at DIV4. (a) Cell viability, (b) [3H] DA uptake, (c and d) Western blot analysis for TH activity, and (e and f) TUNEL staining for apoptotic cells are shown after treatment in DA cultures. Scale bar: 25 *μ*m. Results were obtained from six independent experiments. ^*∗*^*P* < 0.05.

Cell viability, [^3^H] DA uptake, tyrosine hydroxylase (TH) activity, and TUNEL staining assays were performed to inspect the neuroprotective effect of VPA on MPP^+^-induced neurotoxicity in DA neuronal cultures. Different doses of VPA were given after treatment with 10 *µ*M MPP^+^ at DIV4. A cell viability assay showed that MPP^+^ caused approximately 50% neuronal loss compared with the control group 48 h after treatment (Figure 1(a), *P* < 0.05). [^3^H] DA uptake and TH activity were reduced to about 32% of the original level (Figures 1(b)–1(d), *P* < 0.05). TUNEL staining showed that MPP^+^ provoked apoptosis in 35%  ± 8% DA neurons compared with the control group (Figures 1(e) and 1(f), *P* < 0.05). VPA significantly attenuated MPP^+^-induced reduction of cell viability, [^3^H] DA uptake, TH activity, and apoptosis in a dose-dependent manner when compared with the MPP^+^ group (all *P* < 0.05). Thus, VPA conferred protection against MPP^+^ induced toxicity in DA neurons. A VPA concentration of 0.6 mM was chosen as optimal for subsequent studies to avoid a potential toxic side effect at high concentrations.

### 2.2. VPA Reversed MPP^+^-Induced Activation of Mitochondrial Apoptosis Signaling in DA Neurons

**Figure 2:**
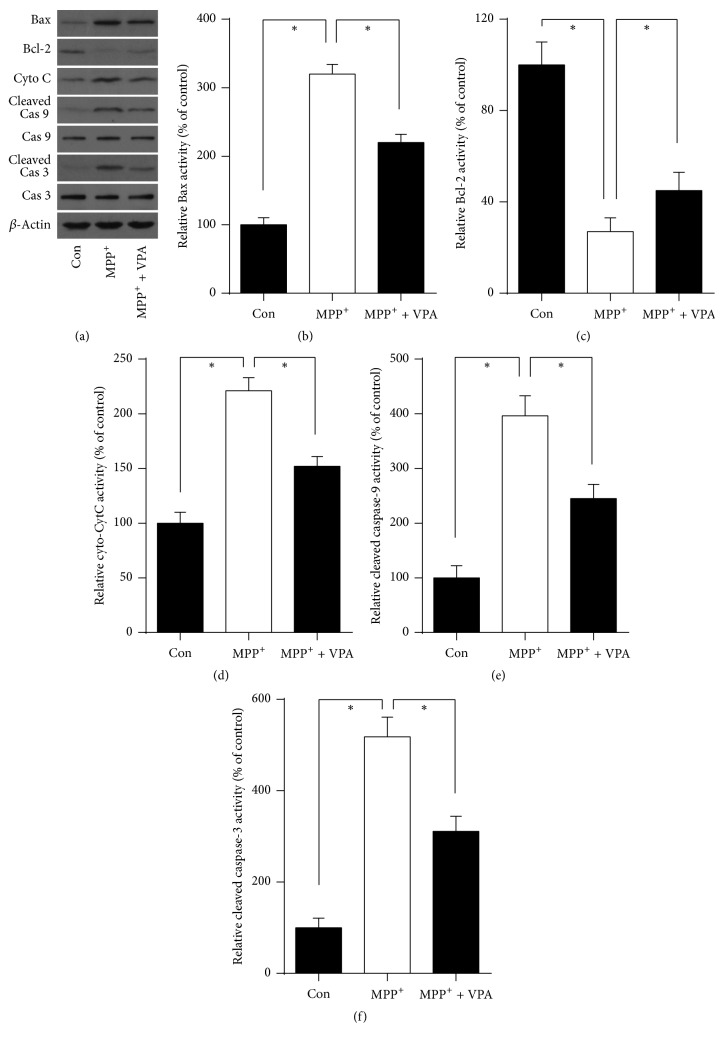
VPA reversed MPP^+^-induced activation of mitochondrial apoptosis signaling in DA neurons. VPA (0.6 mM) was given to cultured DA neurons after treatment with 10 *µ*M MPP^+^ for 48 h at DIV4. (a) Western blot analysis of proapoptotic Bax, antiapoptotic Bcl-2, cytochrome c, caspase-9, and caspase-3, (b and c) quantification of expression of proapoptotic protein Bax and antiapoptotic protein Bcl-2, (d) cytochrome c release in the cytoplasm, and (e and f) relative amount of cleaved caspase-9 and caspase-3. *β*-Actin expression was used as the internal control. Results were obtained from six independent experiments. ^*∗*^*P* < 0.05.

It is well known that the mitochondrial apoptosis pathway plays a key role in cell death and survival. Some apoptosis signaling molecules are indispensable in the process leading to apoptosis. Examples are the Bcl-2 family members Bax (proapoptotic) and Bcl-2 (antiapoptotic), cytochrome c as an essential component of the electron transport chain, and caspase-9 and caspase-3 as executioners of the intrinsic mitochondrial apoptosis pathway. MPP^+^ administration significantly initiated the mitochondrial apoptosis pathway as shown by Western blot analysis (Figure 2(a)). MPP^+^-treatment led to the upregulation of proapoptotic Bax (Figures 2(a) and 2(b)), downregulation of antiapoptotic Bcl-2 expression (Figures 2(a) and 2(c)), enhanced cytochrome c release (Figures 2(a) and 2(d)), and activation of caspase-9 and caspase-3 (Figures 2(a) and 2(e) and Figures 2(a) and 2(f)) (all *P* < 0.05). In contrast, VPA significantly reversed MPP^+^-induced activation of apoptosis signaling by reducing Bax expression (Figures 2(a) and 2(b)), enhancing Bcl-2 expression (Figures 2(a) and 2(c)), reducing cytochrome c release (Figures 2(a) and 2(d)), and reducing caspase cleaving (Figures 2(a) and 2(e) and Figures 2(a) and 2(f)) (all *P* < 0.05).

### 2.3. VPA Upregulated Akt and ERK1/2 Activation and GSK3*β* Phosphorylation following MPP^+^-Treatment of DA Neurons

**Figure 3:**
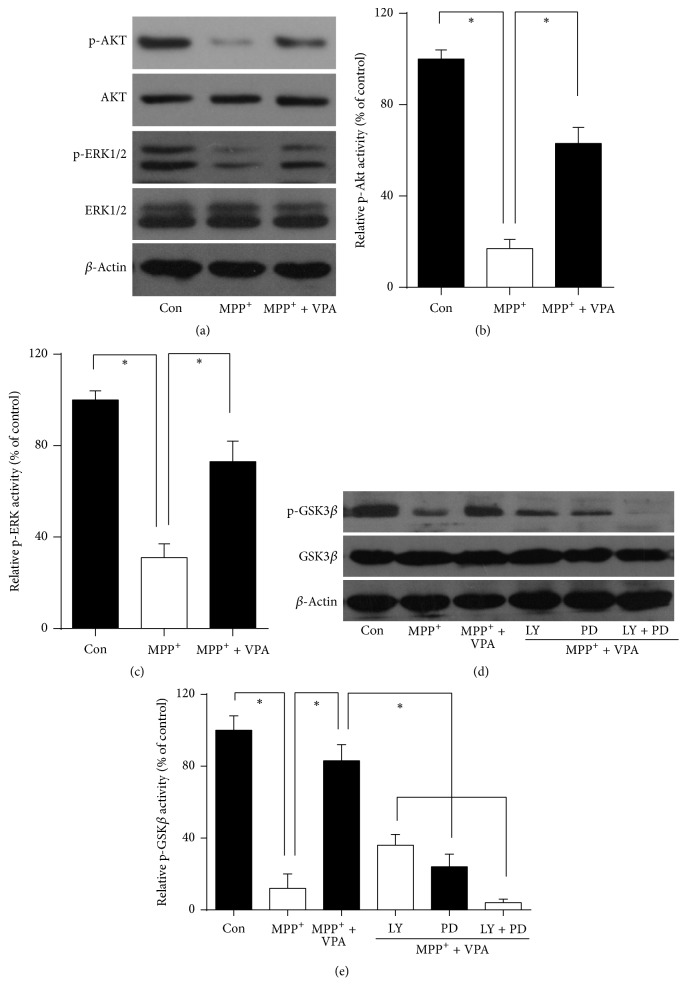
VPA upregulated Akt and ERK1/2 activation and p-GSK3*β* expression after MPP^+^-treatment, whereas inhibitors of PI3K and MAPK pathways counteracted VPA-induced GSK3*β* phosphorylation in DA neurons. VPA (0.6 mM) was given in DA neuron cultures after treatment with 10 *µ*M MPP^+^ for 48 h at DIV4. (a) Western blot analysis of p-Akt, Akt, p-ERK1/2, and ERK1/2 expression. (b) Quantification of Western blot data for relative p-Akt and (c) p-ERK activity. (d) Western blot analysis of GSK3*β* and p-GSK3*β* expression with or w/o PI3K and MAPK pathway inhibitors LY294002 (LY) and PD98059 (PD), respectively. (e) Quantification of Western blot data in (d) for relative p-GSK3*β* activity. The data are from of six independent experiments. ^*∗*^*P* < 0.05.

The phosphoinositide 3-kinase (PI3K) pathway is an extremely important signal transduction system that contributes to many fundamental cellular processes [21]. Together with the mitogen-activated protein kinases (MAPK) pathway that responds to a diverse array of stimuli [22], they both play a key role in determining cell fate. Thus, after confirming the neuroprotective effect of VPA against MPP^+^ insult via reversing activation of mitochondrial apoptosis in DA neurons, we examined whether VPA activated PI3K and MAPK pathways in DA neuron cultures. Akt, the major protein downstream of PI3K and ERK1/2, the key component of MAPK, were analyzed first (Figure 3(a)). Western blot analysis showed that Akt phosphorylation was significantly attenuated after MPP^+^-treatment (*P* < 0.05), but VPA was able to reverse the downregulation of p-Akt (*P* < 0.05) (Figure 3(b)). Moreover, a clear decrease in ERK phosphorylation was observed following MPP^+^-treatment (*P* < 0.05). Again, VPA treatment significantly reversed the effect and increased phosphorylation of ERK (*P* < 0.05) (Figure 3(c)). These results suggested that PI3K and MAPK signaling pathway activation probably both are related to VPA's neuroprotective effects.

Previous studies have shown that GSK3*β* is linked to PI3K and MAPK signaling and is associated with mitochondrial intrinsic apoptosis [20, 23]. Western blot analysis indicated a substantial suppression of GSK3*β* phosphorylation after MPP^+^-treatment compared with the control group (Figures 3(d) and 3(e), *P* < 0.05). VPA administration counteracted suppression of GSK3*β* phosphorylation (Figures 3(d) and 3(e), *P* < 0.05). Furthermore, LY294002, a specific inhibitor of PI3K, and PD98059, a MAPK kinase inhibitor, were used to examine whether the PI3K or MAPK pathway contributed to GSK3*β* inactivation by phosphorylation following VPA treatment (Figures 3(d) and 3(e)). Both inhibitors significantly abolished the effect of VPA on GSK3*β* phosphorylation (Figure 3(e), all *P* < 0.05). When both inhibitors were applied together, almost no GSK3*β* phosphorylation occurred (Figures 3(d) and 3(e)  *P* < 0.05).

### 2.4. Inhibitors of the PI3K and MAPK Pathway Counteracted VPA-Induced Neuroprotective Effects in DA Neurons

**Figure 4:**
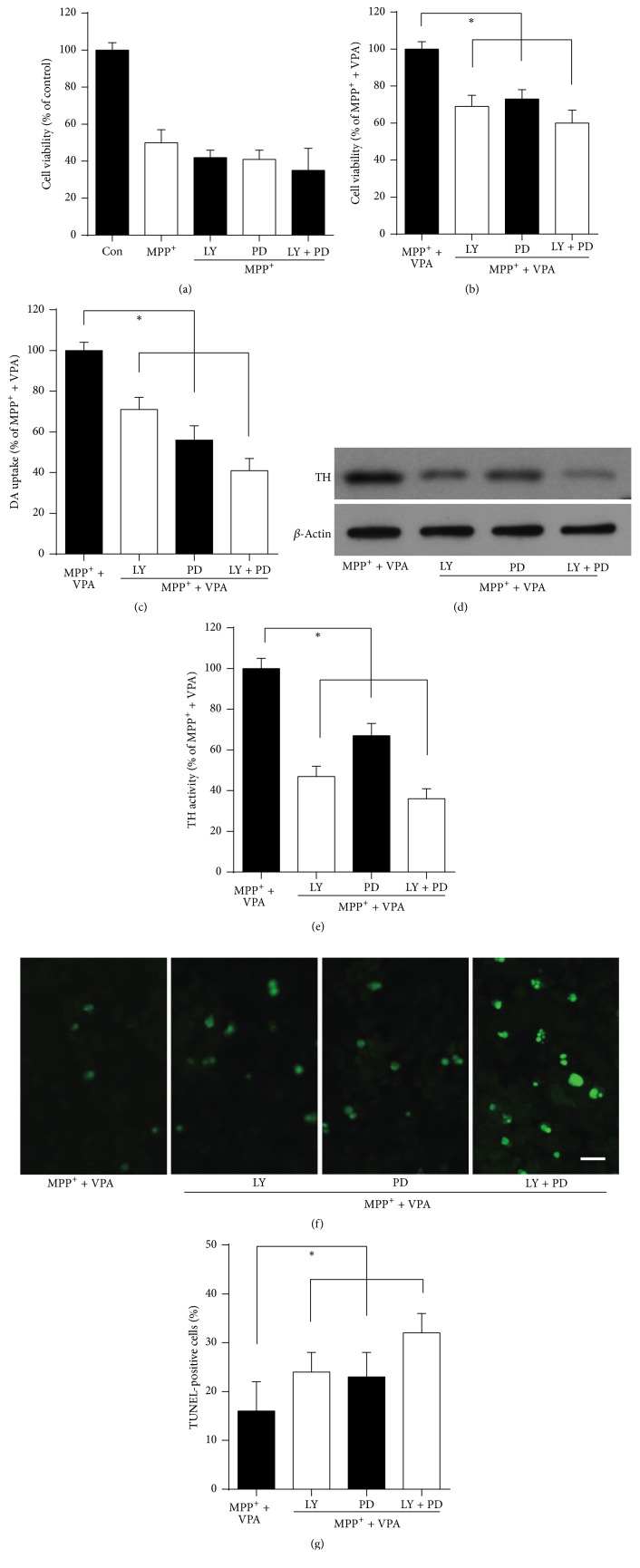
Inhibitors of the PI3K and MAPK pathway reduce the VPA-induced neuroprotective effect in DA neurons in culture. VPA (0.6 mM) was given in DA neuron cultures after treatment with 10 *µ*M MPP^+^ for 48 h at DIV4. (a) Cell viability assay for MPP^+^ plus PI3K and MAPK pathway inhibitors LY294002 (LY) and PD98059 (PD), respectively. (b) Cell viability assay, (c) [^3^H] DA uptake, (d and e) Western blot analysis and quantification for TH activity, and (f and g) TUNEL staining and quantification for apoptotic neurons for MPP^+^/VPA plus LY and PD treatment. Results were from six independent experiments. ^*∗*^*P* < 0.05.

When the PI3K and MAPK pathway inhibitors LY294002 and PD98059 were applied to MPP^+^-treated DA neurons, no effect on reduction of cell viability was observed (Figure 4(a), *P* > 0.05). However, both inhibitors significantly decreased cell viability (Figure 4(b)), impaired DA uptake (Figure 4(c)), reduced TH activity (Figures 4(d) and 4(e)), and increased the number of TUNEL-positive apoptotic neurons (Figures 4(f) and 4(g)) in MPP^+^/VPA-treated DA neurons. Both inhibitors combined further reduced the VPA-induced neuroprotective effect in MPP^+^-treated DA neurons (Figures 4(b)–4(g), all *P* < 0.05).

## 3. Discussion

The present study investigated the neuroprotective effects induced by VPA via the mitochondrial intrinsic apoptosis pathway in a MPP^+^ model of PD. VPA achieved a dose-dependent neuroprotective effect against MPP^+^-induced neurotoxicity in primary dopaminergic neurons as revealed by cell viability, [^3^H] DA uptake, tyrosine hydroxylase (TH) activity, and TUNEL staining assays. Moreover, VPA reversed the apoptosis process initiated by MPP^+^ as shown by analyzing the key mitochondrial apoptosis signaling molecules. For instance, VPA attenuated expression of proapoptotic factor Bax and upregulated antiapoptosis factor Bcl-2 expression. Also, cleavage of the apoptosis executioners caspase-9 and caspase-3 and cytochrome c release were inhibited. Therefore, mitochondrial intrinsic apoptosis pathway appeared to mediate MPP^+^-induced neurotoxicity which was rescued by VPA which shielded dopaminergic neuron in the MPP^+^ model of PD.

PD is a progressive degenerative disorder characterized by the loss of dopaminergic neurons in the substantia nigra pars compacta (SNpc). Patients show no obvious clinical symptoms in the early stages of the disease; however, when the first signs of motor dysfunction begin to appear, at least 50–70% of the dopaminergic neurons are already lost [24, 25]. MPTP, 1-methyl-4-phenyl-1,2,3,6-tetrahydropyridine, is a neurotoxin specific for dopaminergic neurons and is used in classic toxin-induced PD models. It easily permeates the blood-brain barrier and is metabolized to its active form, 1-methyl-4-phenylpyridinium (MPP^+^), by the enzyme monoamine oxidase-B (MAO-B). MPTP causes parkinsonism by selectively damaging dopaminergic neurons and as a consequence leading to the depletion of striatal dopamine (DA) through dopamine transporters (DAT) [26–29]. It mimics the histological and biochemical characteristics of PD by selectively destroying catecholaminergic neurons in the SNpc. Thus VPA contributes greatly to the elucidation of both disease pathogenesis and potential use as neuroprotective therapeutics [13, 16–18, 30–35]. Previous studies demonstrated that MPTP inhibits the mitochondrial complex I, increases production of ROS, leads to *α*-synuclein accumulation, and accelerates dopaminergic cell death [36–39]. By utilizing MPTP in vivo or MPP^+^ in vitro, it has been discovered that SNpc dopaminergic neurodegeneration is associated with the activation of the mitochondrial intrinsic apoptotic pathway [37, 40], which may be a major pathological route in neurodegenerative diseases [41, 42]. Modifications of intrinsic pathway molecules, such as release of cytochrome c, activation of caspase-9 and caspase-3, and regulation of proapoptotic protein Bax and antiapoptotic protein Bcl-2, are confirmed to be directly linked to dopaminergic neuronal death [36, 43, 44]. Therefore, further investigating the mitochondrial intrinsic apoptotic pathway is extremely important for elucidating the etiology of neurodegeneration in PD.

For decades, scientists have tried to find the appropriate agents that protect dopaminergic neurons in PD. There is a spectrum of biomolecules and drugs that claim to have neuroprotective effects against MPTP or MPP^+^ insult in vivo and in vitro. For instance, it has been suggested that active ingredients, such as natural antioxidants, or extracts from traditional Chinese herbs provide neuroprotective effects and harbor antiapoptotic capacities [45–48]. However, besides these recently identified compounds, VPA, as one of the most tolerated and safest antiepileptic and antibipolar disorder drugs, has proven its neuroprotective effects in varies neuronal models [43–46, 49–51]. In PD models, VPA's neuroprotection may associate with anti-inflammatory and antioxidant properties [52, 53] and might be initiated by VPA-induced release of neurotrophic factors from astrocytes or microglia [12, 53]. Additionally, using *α*-synuclein as a PD-marker protein, alterations caused by the neurotoxic challenge could be reverted by treatment with VPA [11]. These findings gathered evidence that VPA provided neuroprotection in various PD models. However, the mitochondrial intrinsic apoptotic molecular pathway, which is an essential signal transduction pathway that determines cell fate, has not received enough attention as a possible target pathway for the neuroprotective effect of VPA in PD models.

It has been demonstrated that VPA activates the lipid kinase phosphatidylinositol 3-kinase (PI3K) and its main downstream targets including prosurvival protein kinases, such as the protein kinase B (PKB)/Akt, the mitogen-activated protein kinases (MAPKs), and other pathways. The PI3K/Akt signaling pathway is indispensably associated and plays a fundamental role in neuronal survival and neuroprotection [54–57]. VPA causes an upregulation of Akt activation via phosphorylation mediated by the PI3K pathway in both in vitro and in vivo models and affects a variety of apoptosis associated molecules and genes [50, 58, 59]. MAPK is another important signal transduction pathway involved in neuronal survival. VPA activates extracellular signal-regulated kinases (ERKs) and promotes neurotrophic effects [50, 60–62]. In our present study, VPA administration significantly upregulated Akt and ERK activation that was impaired by MPP^+^-treatment. Thus, these two pathways may both contribute to the VPA neuroprotective effects. Moreover, GSK3*β* activity, a serine/threonine protein kinase which is regulated through phosphorylation by several kinases, including PI3K and MAPK, was also altered by VPA. Inhibition of Akt and ERK by MPP^+^ causes less GSK3*β* phosphorylation and hence increased activity. VPA treatment, in contrast, restored the inhibitory phosphorylation of GSK3*β* by upregulation of Akt and ERK activity. Furthermore, blocking PI3K and/or MAPK pathway activation using the specific inhibitors LY294002 and PD98059 resulted in decreased GSK3*β* phosphorylation and a fading neuroprotective effect. In summary, it is well established that GSK-3*β* plays a critical role in the CNS and that VPA can inhibit GSK-3*β*'s activity [56, 63, 64]. However, our result demonstrated for the first time that VPA-dependent GSK3*β* phosphorylation is associated with regulation of both Akt and ERK activation, and these pathways are associated with mitochondrial intrinsic apoptosis as part of the neurotoxic MPP^+^ effect in DA neurons in culture.

## 4. Materials and Methods

### 4.1. DA Neuron Culture Preparation

Timed-pregnant BALB/c mice were obtained from the Laboratory Animal Center of the Xiangya School of Medicine, Central South University. All experimental animal protocols and handling procedures were approved by the Institutional Animal Care and Use Committee of the Xiangya School of Medicine, Central South University, in accordance with the National Institutes of Health (NIH) Guidelines for the Use of Laboratory Animals. In brief, postnatal day (P0) rat pups were killed by decapitation and the brains were harvested. The mesencephalic flexure enriched with DA neurons was dissected and the substantia nigra (SN) was isolated and then mechanically dispersed into tissue pieces. Fragment tissues were incubated and dissociated by 0.25% Trypsin/EDTA for 15 min at 37°C in a CO_2_ incubator. Subsequently, DA neurons were centrifuged at 200 g for 5 min and resuspended in Neurobasal medium containing 2% B27 supplement, 2 mM l-glutamine, 100 mg/ml streptomycin, and 100 U/ml penicillin (all from Thermo Fisher Scientific Inc., Waltham, MA, USA). Neurons were plated at a density of 3 × 10^5^ cells/cm^2^ and maintained at 37°C in a humidified 5% CO_2_ jacket incubator. Every two days, half of the culture medium was changed. The cultures survived for seven days in vitro (DIV7) without significant loss of DA neurons, dopaminergic neurons amounted to 15% of total neurons as indicated by immunostaining for tyrosine hydroxylase (TH).

### 4.2. Exposure of DA Neuron Cultures to MPP^+^ and VPA

The neuron culture medium was changed to serum free medium with minimal constitutive activity of kinases, 16 h before experimental treatments as previously described [66]. Our preliminary experiments showed that, at DIV4, 10 *µ*M concentration of 1-methyl-4-phenylpyridinium (MPP^+^; Sigma-Aldrich Chemical Co., St Louis, MO, USA) produces LC50 for DA neurons, which is consistent with previous results [67]. This dose was used for all subsequent studies. After MPP^+^ exposure for 30 min, the cultures were treated with 0.2–1.2 mM VPA (Sigma-Aldrich Chemical Co.) for 48 h.

### 4.3. Cell Viability Assay

MPP^+^- and VPA-treatment effect on cell viability was assessed using a cell viability assay kit (Promega, Madison, WI, USA), according to the manufacturer's instructions. The cell viability assay uses the indicator dye resazurin to measure the metabolic capacity of cells and estimate the number of viable cells. After 48 h, 25 *µ*L of treated cell samples was collected, and then 5 *µ*L of cell viability assay reagent per well was added and the plates were incubated at 37°C for 1 h. The fluorescent signal was recorded by a microplate fluorometer (Thermo Fisher Scientific Inc.) at 560/590 nm.

### 4.4. The [^3^H] DA Uptake Assay

The [^3^H] DA uptake assay was performed following previous studies [68]. Briefly, after being rinsed with warm Krebs–Ringer buffer (KRB; Sigma-Aldrich Chemical Co.), neuron cultures were incubated in 0.5 ml of uptake buffer containing 5 *µ*M dopamine and 20 nM [^3^H] dopamine for 10 min at 37°C. After the assay was stopped by being washed 3 times with ice-cold KRB, the cells were collected and solubilized in 1 M NaOH. Radioactivity was determined by liquid scintillation counting. Nonspecific dopaminergic (DA) uptake determined in the presence of mazindol (10 mM) was subtracted from total uptake to obtain specific DA uptake.

### 4.5. Terminal Deoxynucleotidyl Transferase dUTP Nick end Labeling (TUNEL) Assay

Using an in situ cell death detection kit (Roche, Mannheim, Germany), the level of MPP^+^-induced toxicity and the VPA neuroprotection effect were assessed by observe DNA strand breaks in nuclei, following the manufacturer's instructions. In brief, after treatment with MPP^+^ and VPA, DA neuron cultures were immersed and fixed by freshly prepared fixation solution containing 4% paraformaldehyde in phosphate-buffered saline (PBS, pH 7.4) for 60 min at room temperature. DA neurons were then washed twice in PBS and permeabilized by 0.2% Triton X-100 in PBS for 5 minutes. DA neurons were labeled with fluorescein TUNEL reagent mixture in dark humidified incubator at 37°C for 60 min. After that, slides were reviewed and scored by a Leica fluorescence microscope (Buffalo Grove, IL, USA), and the number of TUNEL-positive (apoptotic) cells was counted. These experiments were repeated six times and the data were summarized as % of control.

### 4.6. Western Blot Analysis

After MPP^+^- and VPA treatment for 48 h, DA neuron cultures were washed with ice-cold PBS for three times and lysed with a lysis buffer (Cell Signaling Technology Inc., Beverly, MA, USA). The protein concentration was measured using a BCA protein assay kit (Thermo Fisher Scientific Inc.). Equal amounts of protein (60 *μ*g/sample) were loaded and separated by 10% SDS-PAGE and transferred to polyvinylidene difluoride (PVDF) membranes (Thermo Fisher Scientific Inc.). Membranes were blocked with 5% nonfat milk solution in* Tris*-buffered saline with 0.1% Triton X-100 (TBST) for 1 h at room temperature and then incubated overnight at 4°C with the primary antibody dilutions in TBST: Akt (1 : 1000), phospho-Akt (1 : 800), ERK1/2 (1 : 1000), phospho-ERK1/2 (1 : 800), GSK3*β* (1 : 1000), and phospho-GSK3*β* (pSer^9^) (1 : 800; all from Cell Signaling Technology Inc.), Bax (1 : 800), Bcl-2 (1 : 1000), cytochrome c (1 : 1000), caspase-3 (1 : 800), and caspase-9 (1 : 800; all from Cell Signaling Technology Inc.). After that the membranes were washed and incubated with secondary antibodies for 1 h at room temperature. Immunoreactivity was detected with Super Signal West Pico Chemiluminescent Substrate (Thermo Scientific, Rockford, IL, USA). Image J image analysis software (National Institute of Heath, Bethesda, MD, USA) was used to quantify the optical density of each band. The activation of Akt and ERK1/2 is presented as the ratio of phosphorylated kinase bands to total kinase bands. The activation of caspase-9 and caspase-3 is presented as the ratio of cleaved bands to the total bands.

### 4.7. Statistical Analysis

Statistical analysis was performed using the SPSS 18.0 statistical software package (SPSS Inc., Chicago, IL, USA). The data were presented as mean ± SD, and one-way ANOVA was used for statistical analyses followed by the Student–Newman–Keuls test. For all tests, the level of statistical significance was set at *P* <0.05.

## 5. Conclusions

Our results provided evidence that the antiepileptic drug VPA has neuroprotective and antiapoptotic effects in an in vitro PD model. VPA's antiparkinsonian capability is likely associated with its effect on GSK3*β* phosphorylation through Akt and ERK activation as part of mitochondrial intrinsic apoptosis signaling. Although clinical studies of VPA's effects on PD started in the 1970s, these studies focused only on a small number of patients with rather short-term follow-up times. Thus, these studies' conclusions are still questionable [47, 48, 65]. Given recent data on the neuroprotective effect of VPA, it is still a valid therapeutic candidate for clinical treatment of PD but larger and well controlled clinical trials are in urgent need.
